# Authoritative Filial Piety Rather than Reciprocal Filial Piety Mediated the Relationship Between Parental Support, Career Decision Self-Efficacy, and Discrepancies Between Individual-Set and Parent-Set Career Goals

**DOI:** 10.3390/bs15081135

**Published:** 2025-08-21

**Authors:** Shanshan Guan, Fanrong Meng, Chenggang Wu

**Affiliations:** 1 Key Laboratory of Multilingual Education with AI, School of Education, Shanghai International Studies University, Shanghai 202620, China; guanss0123@163.com; 2 Center for Comparative Study of Global Education, School of Education, Shanghai International Studies University, Shanghai 202620, China; 0233100636@shisu.edu.cn; 3 Institute of Language Sciences, Shanghai International Studies University, Shanghai 202620, China; 4 Shanghai Key Laboratory of Brain-Machine Intelligence for Information Behavior, Shanghai International Studies University, Shanghai 202620, China

**Keywords:** career goals, parental support, career decision self-efficacy, discrepancies, filial piety

## Abstract

Although a wealth of research has examined the predictors influencing the discrepancies between individual-set and parent-set career goals (DBIPCG), investigations grounded in collectivist cultural perspectives remain relatively scarce. Within collectivist societies, filial piety holds profound cultural significance. Drawing on a dual filial piety framework encompassing reciprocal filial piety (RFP) and authoritative filial piety (AFP), this study aims to explore the interconnections among parental support, self-efficacy in career decision-making, dual filial piety orientations, and DBIPCG. The results indicated that parental support was negatively associated with DBIPCG. By contrast, self-efficacy in career decision-making did not predict DBIPCG directly. Instead, self-efficacy indirectly influenced DBIPCG, an effect mediated specifically by AFP rather than RFP, Furthermore, AFP was found to mediate the link between parental support and DBIPCG. These findings underscore the role of parental support in minimizing differences in career goal formation between generations and highlight the potentially adverse implications of AFP in exacerbating such discrepancies.

## 1. Introduction

Career goals are fundamentally significant for young people, especially college students, as they influence actual career-related decisions (Lent et al., 1994), serve as foundational elements for future professional success (Schoon & Polek, 2011), and contribute to the development of vocational identity and readiness (Kemph, 1969). Disagreements between parents and children regarding career goals have drawn considerable scholarly attention due to their potential to hinder key developmental processes in young people, including participation in career-oriented tasks such as exploration and decision-making (Sawitri et al., 2021). According to Anderson and Mounts (2012), navigating misalignments with parents about career direction often results in prolonged disputes and forced adjustments of personal aspirations (Bedford & Yeh, 2019). Research in career development suggests that adolescents from collectivist cultures prioritize their parents’ expectations and preferences. Specifically, they are more likely to pursue career paths aligned with parental advice instead of their interests (Tang, 2002). Disagreements and misalignments in career expectations may not only hinder the career development of young individuals (P.-W. W. Ma & Yeh, 2005) but lower the quality of parent–child relationships, especially in situations where children forgo their ambitions out of a sense of filial obligation (Onifade et al., 2016; Tang, 2002). Filial piety, as a culturally embedded model of parent–child relations, also reflects how children interpret and respond to parental expectations, Existing research has also suggested that filial piety is critical in shaping the sense of purpose of life of individuals, perceived well-being, and career orientation (Sun et al., 2023; Wei et al., 2019; Yao et al., 2015). Therefore, examining how filial piety relates to parent–child tensions around career decision-making is critically important (in this research, China).

Contemporary Chinese youth navigate career choices within a complex interplay of cultural traditions, rapid socioeconomic transformation, and globalized influences. As Lian and Feng (2021) note, younger generations increasingly exercise autonomy in career decisions, prioritizing personal interests, skills, and prospects amid a diversifying labor market. Yet this autonomy operates within structural constraints: hypercompetitive job markets, state policy shifts (e.g., “Common Prosperity” initiatives), and heightened economic uncertainty postpandemic intensify pressures on both youth and parents (Zhao & Tong, 2025). Crucially, parental expectations remain deeply embedded in cultural norms of filial piety but are dynamically reshaped by contemporary anxieties about social mobility and economic security (H. Song, 2021). Parents often strategically encourage “secure” careers (e.g., civil service, tech sectors) to mitigate perceived risks—a pragmatic response to societal volatility that transcends purely traditional motivations. This evolving negotiation between individual agency and familial influence underscores the need to contextualize filial piety not as a static cultural relic, but as an adaptive framework mediating modern career tensions.

Within this landscape, authoritative filial piety (AFP) and reciprocal filial piety (RFP) function as fluid psychological schemas rather than timeless cultural monoliths. Current scholarship (e.g., Bedford & Yeh, 2021) emphasizes that filial norms are continually reinterpreted through interactions with structural forces: AFP’s emphasis on hierarchical obedience may amplify under labor market precarity, as parents prioritize stability over passion, while RFP’s focus on mutual care increasingly contends with youths’ exposure to global individualistic ideals (e.g., self-actualization, work–life balance). As youth reconcile parental expectations rooted in collectivist values with neoliberal career discourses, filial piety manifests in hybridized forms—sometimes reinforcing intergenerational alignment, other times exacerbating goal conflicts. This study thus positions AFP and RFP as sociocognitive adaptations to the unique Chinese institutional environment, where filial norms are both prescribed by cultural heritage and reconfigured by economic pressures, policy landscapes, and cross-cultural ideational flows.

To empirically investigate these dynamics, we examine discrepancies between individual-set and parent-set career goals (DBIPCG) through a filial piety lens, extending Sawitri et al.’s (2021) foundational work on parent–youth goal misalignment. Their 15-item instrument measuring perceived gaps between self-set and parent-set career goals provides a critical framework yet lacks explicit theorization of cultural mediators in collectivist societies. Integrating social cognitive career theory (SCCT), we argue that filial piety orientations (AFP/RFP) shape how adolescents internalize parental career expectations, thereby modulating career decision self-efficacy. Specifically, we propose that AFP—emphasizing duty-bound compliance—may intensify perceived discrepancies and undermine self-efficacy when youth face conflicting goals, whereas RFP—premised on mutual respect—could buffer such tensions by fostering collaborative negotiation. This approach not only addresses critiques of cultural determinism but illuminates the psychological mechanisms through which macrostructural forces become lived experiences in career development.

## 2. Theoretical Framework

### 2.1. Social Cognitive Career Theory (SCCT)

Widely applied in career development research, SCCT is built upon three key theoretical foundations: self-efficacy theory (Bandura, 1977), career self-efficacy theory (Hackett & Betz, 1981), and social cognitive theory (Bandura, 1986). SCCT (Lent et al., 1994) is based on the idea that “personal and environmental factors contribute to distinct learning experiences that help shape self-efficacy and expectations of outcomes, which subsequently affect the formation of academic and career interests, goals and behaviors” (Kantamneni et al., 2018). SCCT integrates various psychological and contextual elements contributing to career-related decision-making processes (Lent et al., 1994). Lent et al. (2000) emphasized that effective career development frameworks should consider various contextual factors, including economic status, environmental influences, gender, culture, peer dynamics, and particularly parental involvement (Lent et al., 2000).

The SCCT framework consists of two phases of theoretical construction. The first phase emphasizes three key cognitive mechanisms: self-efficacy beliefs, outcome expectations, and the formulation of personal goals that guide individuals through their career development journeys (Lent et al., 1994, 2000). Among these, self-efficacy and outcome expectations function as mediating constructs within the decision-making process, reflecting how individual traits and situational opportunities, particularly those derived from learning experiences, jointly influence career planning (Lent & Brown, 2006). In the second phase, factors such as personal inputs and contextual background affordances, including family and ethnicity, are considered, as they can impact career-related decisions (Wilk et al., 2018). Within this framework, context-specific factors, often labeled as barriers (e.g., discouragement, limited access), have significantly correlated with individuals self-efficacy and behavioral intention patterns (Lent et al., 2010).

Factors such as socioeconomic status, home environment, family resources, and parental support and influence play a crucial role in shaping learners’ career decisions, serving as supportive elements and potential barriers in their decision-making process (Van Wyk, 2011). Bandura suggests that learners often lack direct control over the family circumstances and parental viewpoints that impact their daily experiences, as parental influences typically affect children’s behavior through intermediary self-regulatory processes rather than through direct interactions (Bandura, 2002). Therefore, children are inclined to pursue career paths and make job choices that align with their parents’ expectations and preferences, selecting environments that resonate with those familial demands (P. K. Wells, 2013). Research indicates that parents substantially impact how their children view specific careers and professions. (Samsuri et al., 2016). Their parents’ own aspirations frequently shape children’s career ambitions, as parents typically desire for their children to pursue a profession that offers a better quality of life than they experienced (Edwards & Quinter, 2011).

Numerous researchers have employed SCCT to examine how students’ career and educational development unfold over time (Gibbons & Borders, 2010). While most empirical work has emphasized the first phase of the SCCT framework, subsequent stages—incorporating personal characteristics, situational influences, and job-related satisfaction—remain relatively underexplored (Lent et al., 2000). Understanding these later-stage elements may be crucial for uncovering key discrepancies in career-related decision-making among undergraduate populations.

### 2.2. Goal-Setting Theory

Rooted in the framework introduced by G. Latham and Yukl (1976), goal-setting theory posits that consciously formulated goals significantly contribute to the regulation of individual behavior (G. Latham & Yukl, 1976). Within this theoretical context, the degree to which individuals commit to their goals is vital for realizing desired achievements (Hollenbeck et al., 1989). Research also supports that sustained commitment is positively associated with persistence (Klein et al., 2012), fostering greater endurance when pursuing complex or demanding objectives. Goal-setting theory (Locke & Latham, 1990) posits that individuals often demonstrate enhanced performance when their objectives are well-defined, challenging, and realistic. Locke and Latham’s empirical work reinforces this by showing that setting ambitious and articulated goals is more effective in driving performance than vague or easily attainable ones (Locke & Latham, 1990). Additionally, goals serve psychological functions by addressing intrinsic needs, such as personal development, accountability, acknowledgment social inclusion, and perceived safety, as outlined by Steers and Porter (Steers & Porter, 1974, p. 19). Rather than relying on external motivators, setting internalized goals provides strategic benefits, including simplicity and practical application (G. P. Latham & Locke, 1979). Put differently, individuals are typically more inclined to pursue outcomes they have personally defined as opposed to reacting to external demands or imposed expectations (G. Latham, 2012).

Existing research has identified two primary factors that motivate individuals to pursue challenging career aspirations. The first of these factors is self-efficacy (Bandura & Locke, 2003). Individuals with stronger beliefs in their capabilities tend to demonstrate greater persistence in working toward their career objectives over extended periods (Locke & Latham, 2002). Interest is the second factor (Donovan, 2009), as an individual’s level of engagement with a task often determines their willingness to seek out demanding opportunities (Donovan, 2009). Beyond clarity and task difficulty, the goal-setting model also incorporates components such as involvement in goal definition, feedback mechanisms self-belief, and personal interest. It emphasizes that this framework can be broadly applied in contexts where individuals or collectives influence outcomes (Locke & Latham, 2006). Goal-setting theory provides a valuable lens for understanding how multiple elements shape students’ career goal development and the steps they take to attain those goals, primarily through goal-directed behavior that supports employability enhancement (Clements et al., 2016). In light of this framework, the present study explores the factors contributing to discrepancies between individual-set and parent-set career goals (DBIPCG), drawing on insights from goal-setting theory.

## 3. Literature Review

### 3.1. Dual Filial Piety and Career Goal-Setting in Contemporary Contexts

Filial piety—a multidimensional construct encompassing cognitive, emotional, and behavioral expressions toward parents (Wan et al., 2024)—remains central to understanding career goal-setting in East Asia. However, contemporary scholarship rejects static interpretations, emphasizing instead how filial norms dynamically evolve amid socioeconomic transformations (Bedford & Yeh, 2021). Bedford and Yeh’s dual framework distinguishes reciprocal filial piety (RFP), characterized by emotional closeness and mutual respect, from authoritative filial piety (AFP), which prioritizes hierarchical obedience and deference to parental authority. Crucially, these orientations do not operate in cultural isolation. They are continuously reinterpreted through interactions with structural forces: hypercompetitive labor markets, state policy shifts (e.g., skills-focused “Made in China 2025” initiatives), and youths’ exposure to global individualistic ideals (e.g., self-actualization, work–life balance). This reconceptualization positions AFP and RFP as adaptive schemas that mediate between traditional collectivist values—where parents actively shape children’s career goals (Tynkkynen et al., 2010)—and contemporary pressures. Adolescents increasingly navigate tensions between personal aspirations, parental expectations, and external constraints (Cross et al., 2000), with filial piety serving as a psychological lens through which these conflicts are internalized (Leong et al., 2011).

### 3.2. Parental Support, Expectations, and Filial Piety as Mediating Systems

Parental career support—encompassing both practical guidance and emotional reinforcement—enhances adolescents’ career decision self-efficacy and goal clarity (Alliman-Brissett et al., 2004; Constantine et al., 2005). However, parental expectations introduce complexity. Defined as anticipatory beliefs about children’s career achievements (Y. Ma et al., 2018), their impact hinges on contextual factors: alignment with youth aspirations, labor market volatility, and the mediating role of filial piety (Pinquart & Ebeling, 2020). In China, filial norms intensify expectation internalization, often compelling youth to prioritize parental standards over personal interests (Hou & Leung, 2011; Leung et al., 2011). Yet current research underscores that this process is neither uniform nor deterministic. AFP, emphasizing compliance, may amplify intergenerational conflict when parental expectations clash with youth autonomy or market realities (e.g., declining prestige of traditionally “secure” careers). Conversely, RFP’s mutual respect facilitates collaborative negotiation, potentially aligning goals with contemporary socioeconomic demands (Liu et al., 2015). Critically, both pathways are moderated by macrostructural shifts: postpandemic economic uncertainty heightens parental anxiety, reinforcing AFP-driven interventions, while digital globalization exposes youth to alternative success models, complicating RFP’s emphasis on familial reciprocity. This study thus examines how AFP and RFP distinctly mediate the relationship between perceived parental expectations and career goal discrepancies—a gap in the extant literature.

### 3.3. Parent–Child Interaction Dynamics in Goal Formation

Career goal-setting emerges from iterative negotiations between adolescents’ internal drivers (e.g., self-efficacy; Xiao et al., 2023) and external influences, with parental involvement playing a pivotal role (He & Yao, 2018). Productive interactions involve autonomy-supportive behaviors—parents offering resources while respecting youth agency (Ryan & Deci, 2018)—which foster competence and goal congruence (Feldman, 2007; Gagnon et al., 2019). Conversely, controlling tactics (e.g., guilt-induction, threats) undermine autonomy, exacerbating goal discrepancies and decision-making difficulties (Wang et al., 2007; Xu, 2012). Filial piety fundamentally shapes these dynamics: AFP legitimizes parental control as cultural obligation, risking youth disempowerment when expectations ignore market realities (e.g., gig economy careers). RFP, by contrast, encourages dialogue, allowing families to adapt goals to contemporary constraints (e.g., balancing prestige with employability in AI-disrupted fields). Misalignments often reflect divergent responses to structural change: parents may prioritize stability amid economic precarity, while youth weigh global opportunities (Rutherford, 2015). The dual filial framework illuminates why such discrepancies arise: AFP’s rigid hierarchy impedes compromise, whereas RFP’s relational flexibility enables coconstruction of hybrid goals. This study addresses a critical void by investigating how AFP and RFP differentially mediate the link between parental interaction styles (support/control) and career goal inconsistencies—ultimately shaping career decision self-efficacy.

### 3.4. The Present Study

This study investigates career goal discrepancies between Chinese youth and their parents within a rapidly evolving sociocultural landscape. Contemporary China is characterized by hypercompetitive labor markets, state policy shifts, and youths’ growing exposure to global individualistic ideals—factors that dynamically reshape filial piety expressions and intergenerational negotiations over career planning (Lian & Feng, 2021; Zhao & Tong, 2025). Against this backdrop, we examine how authoritative filial piety (AFP) and reciprocal filial piety (RFP) mediate relationships among parental support, career decision self-efficacy, and discrepancies between individual-set and parent-set career goals (DBIPCG). Drawing on Sawitri et al.’s (2021) validated 15-item DBIPCG scale, we integrate social cognitive career theory (SCCT) and goal-setting theory to unpack these dynamics. SCCT underscores contextual influences—including parental expectations and labor market volatility—on career development (Lent et al., 1994; Wilk et al., 2018), while goal-setting theory highlights self-efficacy’s role in sustaining commitment to self-defined goals (Locke & Latham, 2002). Crucially, parental support is theorized to reduce DBIPCG by fostering youth autonomy (Gagnon et al., 2019). However, in the Chinese collectivist milieu, filial piety may reconfigure this relationship: AFP’s hierarchical compliance may amplify discrepancies when youths confront tensions between parental expectations and personal aspirations amid economic precarity, whereas RFP’s mutual respect may buffer such tensions through collaborative adaptation (Liu et al., 2015; Bedford & Yeh, 2021). To address gaps identified in the literature, we propose:

Research Questions:

RQ1: How are parental support, career decision self-efficacy, and dual filial piety associated with DBIPCG?

RQ2: Do parental support and career decision self-efficacy predict DBIPCG?

RQ3: Does dual filial piety mediate relationships among parental support, career decision self-efficacy, and DBIPCG?

This study adopts a confirmatory approach grounded in social cognitive career theory (SCCT) and Goal-Setting Theory. Based on these theoretical foundations and previous empirical findings, we formulate and test specific directional hypotheses to examine the mediating roles of dual filial piety in career goal discrepancies:

**H1.**  *Parental support is negatively associated with discrepancies between individual-set and parent-set career goals (DBIPCG)*.

**H2.**  *Career decision self-efficacy is negatively associated with DBIPCG*.

**H3.**  *Authoritative filial piety (AFP)—but not reciprocal filial piety (RFP)—mediates the relationships between (a) parental support and DBIPCG and (b) career decision self-efficacy and DBIPCG. Specifically, AFP is expected to exert positive indirect effects in these pathways, reflecting its role in exacerbating goal conflicts when youth internalize parental expectations as obligations*.

Unlike exploratory studies that aim to identify patterns or generate hypotheses, this research is theory-driven and designed to test specific mechanisms derived from SCCT and the dual filial piety model.

## 4. Methods

### 4.1. Participants

Using a convenience sampling method, we recruited 250 Chinese college students, including 106 males, with an average age of 20.4 years (SD = 1.8). Most participants were undergraduates (N = 213), while the remainder were postgraduates, including 35 Master’s students and 2 doctoral students. The majority of participants were aged 19 (23%) and 20 (33%), with smaller proportions at 21 years (12%), 18 years (8.7%), 22 years (8.7%), 23 years (5.9%), 24 years (5.1%), and 25 years (1.6%). The remaining age groups (17 years and 27 years) each accounted for less than 1% of the sample. Participants represented diverse academic disciplines, with distribution as follows: philosophy (N = 3), economics (N = 38), law (N = 1), education (N = 35), literature (N = 35), science (N = 20), engineering (N = 65), management (N = 51), and art (N = 2).

To ensure the representativeness of the sample and the generalizability of the findings, this study adopted a diversified sampling strategy by selecting student participants from ten universities across China. These institutions were deliberately chosen to reflect variation in geographical location, institutional type, and educational tier. Specifically, the selected universities span different regions, including eastern China (3 universities), central China (2 universities), western China (2 universities), and northeastern China (3 universities). They also represent a range of institutional categories, such as comprehensive universities (3 universities), science and engineering institutions (2 universities), teacher training universities (1 university), international language study universities (1 university), and vocational colleges (3 colleges). Furthermore, both “Double First-Class” universities (5 universities) and nonelite institutions, including ordinary undergraduate (2 universities) and higher vocational colleges (3 colleges), were included. This diversity in the sample enhances the heterogeneity of the data and strengthens the external validity and explanatory power of the research findings.

### 4.2. Measurement

Following the section on individual demographic information, the questionnaire consisted of four aspects. The first aspect measured DBIPCG, which was obtained from a study by Swaitri et al. (Sawitri et al., 2021). The items covered ability, choices, and enthusiasm (Sawitri et al., 2021). Participants were required to evaluate 15 items (five items in per dimension) on a Likert scale ranging from 1 (strongly disagree) to 6 (strongly agree). The second aspect measured parental support using 12 items on a 5-point scale (Wang et al., 2007). The third aspect assessed career decision self-efficacy using 6 items on a 10-point scale (Hampton, 2005). The final aspect measured the dual filial piety using 16 items on a 6-point scale (Yeh, 2004). The corresponding author translated the original scales in the DBIPCG; the translated items were then back translated by the first author and then checked by an English major postgraduate student. Consensus was reached to determine the translation’s accuracy and ease of comprehension. The four scales had adequate reliability (see Table 1 for internal consistency reliability). We also performed confirmatory factor analysis on the scales and found all scales had appropriate construct validity (see Table 2 for more information, with χ^2^/df < 5, CFI > 0.9, TLI > 0.9, and SRMR < 0.09 indicating a good model fit).

### 4.3. Procedure

From February to April 2024, we employed wjx, a popular and easy-to-use platform, to distribute online questionnaires to potential participants in China. The wjx (www.wjx.com) has professional social connections with young Chinese people, especially college students. Therefore, past research has deployed it to facilitate data collection (Xiao et al., 2023). Given the difficulty in accessing a large sample of college students in China, this study employed a convenience sampling method, commonly used in social psychology research (Xiao et al., 2023; Zhang et al., 2023). We first sent the scale to teachers and managers in the ten schools, and then these teachers and managers distributed the questionnaire widely to their students. Finally, 250 responses were received and analyzed.

### 4.4. Data Analyses

First, we present descriptive statistics (means and standard deviations) for all variables under investigation. Second, correlation analyses were conducted to examine the relationships among the variables. With regard to the mediation analysis, following the hypothesized model, we used JASP (Love et al., 2019), which interfaces with the lavaan package in R (Rosseel, 2012), to estimate indirect effects. Bootstrapping was employed to compute the indirect effect, and bias-corrected percentile confidence intervals were used to assess the significance of the mediation effects (Biesanz et al., 2010).

## 5. Results

### 5.1. Descriptive Statistics

The DBIPCG was moderately pronounced, indicating that participants in our study encountered conflicts regarding their career goals with their parents. Regarding career decision self-efficacy, participants exhibited a relatively strong sense of confidence, with an average score of 6.27 out of 10. Moreover, participants indicated a moderate perception of parental support and reported experiencing average levels of reciprocal and authoritative filial piety (see Table 1 for more details).

### 5.2. Correlation

The strong correlations among the three dimensions (ability, choice, and enthusiasm) of DBIPCG supported the theoretical validity of the scale (Sawitri et al., 2021). While self-efficacy in making career decisions showed no significant relationship with DBIPCG, it positively correlated with parental support and filial piety. Notably, parental support was associated with both reciprocal and authoritative sense of filial duty. Importantly, in addition to parental support, authoritative filial piety, rather than mutual filial respect, was found to be associated with the DBIPCG, suggesting a potential divergence in the roles of these two forms of filial piety in influencing such discrepancies. (See Table 3).

### 5.3. How Filial Piety Mediated the Associations Among Career Decision-Making Self-Efficacy, Parental Support, and Gaps Between Self-Set and Parent-Set Career Goals

Parental support exhibited a direct negative impact on all aspects of DBIPCG, including ability, choices, and enthusiasm (see Table 4). Importantly, self-efficacy in career decisions was identified as an indirect mediator in predicting DBIPCG, mediated by authoritative filial piety (AFP). Specifically, self-efficacy was fully mediated by AFP, such that the direct effect of self-efficacy on the DBIPCG was not significant. However, the influence of career decision self-efficacy on DBIPCG was established by considering the mediator of AFP. Moreover, it was observed that AFP, rather than reciprocal filial piety (RFP), acted as a mediator within the association linking parental support and DBIPCG in ability, choices, and enthusiasm.

## 6. Discussion

Rooted in a collectivist culture that places significant emphasis on filial piety, the present study explores the relationships among parental support, decision-making self-efficacy, dual filial piety, and discrepancies between individual-set and parent-set career goals (DBIPCG). The results confirmed the associations among DBIPCG, parental support, and authoritative filial piety (AFP). Although no significant link was observed between career decision-making self-efficacy and DBIPCG, positive correlations were identified between parental support and filial piety. Specifically, parental support showed significant associations with reciprocal and authoritative forms of filial piety. Moreover, authoritative filial piety (AFP), rather than reciprocal filial piety (RFP), was found to have a prominent influence on DBIPCG. These findings suggest that the two forms of filial piety may exert distinct effects on forming such discrepancies.

### 6.1. The Role of Parental Support and Self-Efficacy in Career Decision-Making in Discrepancies Between Individual-Set and Parent-Set Career Goals

From SCCT, contextual factors, such as personal career network contacts, are essential in promoting career development (Lent et al., 1994). Parental support, as a part of personal career network contact from essential others, showed a positive association with self-efficacy in career-related decision-making (Kush & Cochran, 1993), career interests (Lapan et al., 1999), and career adaptability (Salim et al., 2024). For example, career adaptability, as an individual’s preparedness to handle unexpected obstacles arising from the continuously changing external environment, can be enhanced with parental and teacher support (Y. Song et al., 2022). The present study extended previous studies by showing that parental support was negatively associated with DBIPCG. This negative link between parental support and DBIPCG is attributed to the fact that if parents provide support, they could also align with their children’s career path choices, abilities, and enthusiasm. Moreover, parents show their support by allowing and promoting their children’s autonomy, facilitating their confidence in career decisions. This is consistent with the favorable association between parental support and confidence in career decision-making.

However, confidence in decision-making in career decisions appeared not to be directly related to DBIPCG, which did not agree with the proposed hypothesis or previous studies (Akosah-Twumasi et al., 2018). For example, a recent review showed that higher career congruence between parents and children was related to increased self-efficacy. However, much of this positive association linking decision-making confidence and perceived professional congruence between parents and their children was observed in Indonesian high school students (Sawitri & Creed, 2017). This study, by contrast, investigated the association between confidence in career-related decisions and DBIPCG within Chinese college students. The different educational contexts, and more importantly, different age groups, might contribute to the inconsistent findings. Specifically, college students, as they enter their twenties, can independently evaluate their self-efficacy in career decisions. Therefore, there could be a weak connection between career decision-making confidence and DBIPCG. Despite the limited direction prediction of self-efficacy on DBIPCG, self-efficacy in career decision-making could predict DBIPCG via the mediation of AFP. Moreover, parental support could also indirectly predict discrepancies between career goals set by individuals and those set by their parents via the mediation of AFP rather than RFP, which would be discussed in the next section.

### 6.2. The Mediation of AFP Rather than RFP

Filial piety (xiao) is an essential virtue that children should respect, concern, and bring glory to their parents (Chen, 2014). Respect for parents is established by a distinct societal tradition that originates from Confucianism, which emphasizes the authority of parents and the family’s hierarchical structure according to generations and ages. Filial piety has been transformed to adapt to new social and parental relationships (Li et al., 2014). Based on this transformation and potential different effects of filial piety, a new filial piety theoretical framework, namely the dual filial piety model, differentiated two forms of filial respect: reciprocal filial piety (RFP) and authoritarian filial piety (AFP) (Yeh, 2004). RFP characterizes a situation in which offspring show their care, respect, and love towards their parents out of their gratitude for being raised and loved by their parents. This filial piety is horizontal, indicating an equal status of parents and children. By contrast, AFP is a negative filial devotion in which children are compelled to comply with their parents’ directives and requests due to vertical interaction and pressure from the family’s social norm of hierarchical structure. The obedience, in most cases, is accompanied by children’s sacrifices. For example, RFP was positively related to extraversion, openness, agreeableness, and conscientiousness in the Big Five personality. In contrast, AFP was negatively associated with extraversion, openness, and conscientiousness but positively associated with neuroticism (Yeh & Bedford, 2003). Moreover, AFP was also negatively correlated with conventionalism and submission to authority, indicating that individuals with high AFP were likelier to adhere to social conventions and obey their superordinate order. One recent study further showed a positive link between RFP and academic performance and a negative relationship between AFP and academic performance, extending the previous studies on personality and filial piety to educational settings. Moreover, there was a positive association between RFP and subjective wellbeing; no relationship was found between AFP and subjective wellbeing, indicating a possible divergence of AFP and RFP (Guo et al., 2022).

The current study strongly demonstrated the disparities between AFP and RFP regarding their mediating roles among personal support, career decision-making self-efficacy, and DBIPCG. It was found that AFP, rather than RFP, mediated the relationships among these factors. Moreover, along with the positive association between AFP and DBIPCG, the mediation of AFP can be explained by the assumption that in a family hierarchy where AFP is emphasized, children are often required to comply with their parents’ instructions and fulfill their parents’ expectations and requirements, even at the expense of their interests. Hence, DBIPCG was strongly linked to AFP. Furthermore, in a vertical parent–child relationship pattern, parental support and self-efficacy in career decisions contributes to an increase in DBIPCG. The greater the support their parents provide, the more discrepancies children tend to encounter, as they feel compelled to meet their parents’ expectations for external rather than internal motivations. AFP also mediated the relationship between self-efficacy in career decision-making and DBIPCG, suggesting that AFP altered the direction of self-efficacy’s impact on DBIPCG. In the case of children with high AFP, their self-efficacy in career decision-making could act as a facilitator for DBIPCG. These findings imply that, in practical terms, to reduce DBIPCG, providing parental support and enhancing self-efficacy in career decision-making are crucial, but more importantly, establishing an adaptive and functional concept of filial piety is highly necessary. Instead of AFP, RFP should be established through a horizontal and close parent–child relationship emphasizing equality, respect, and gratitude.

This study found that AFP played a mediating role in parental support, career decision-making self-efficacy, and DBIPCG, while RFP did not play a similar mediating role. This result reveals the evolving trend in contemporary Chinese parent–child relationships: although modern young people have more autonomy in career choices, traditional AFP still has an important impact on the interaction between parents and children and career decisions. With the rapid development of society, parents still play a key role in their children’s career decisions, but this role is increasingly guiding and supporting rather than simply controlling (H. Song, 2021). In this context, AFP can provide parents with a structured framework to help them maintain a certain influence in supporting their children’s career choices, especially in setting career goals. In contrast, although RFP emphasizes the reciprocal relationship between parents and children, it may be difficult for it to play an effective mediating role in the process of career goal setting because it focuses more on economic returns and two-way dependence.

This finding is of great significance to the understanding of career development in the context of contemporary China, especially revealing the complex interactive relationships among parental expectations, cultural traditions, and modern career choices in parent–child relationships. Based on the findings of this study, several practical implications can be drawn for parents, educators, and children. For parents, it is essential to reflect on their relationship with their children from the perspective of filial piety. Chinese parents should recognize the importance of building a relationship based on equality and mutual, unconditional respect rather than authoritarian expectations. This approach can help prevent the emergence of AFP, which the present study identified as a mediating factor between parental support and DBIPCG. Under the influence of AFP, increased parental support may paradoxically hinder the development of a shared vision in career decision-making, leading to greater emotional strain on the child. For educators and counselors, it is crucial to consider the quality of the parent–child relationship when providing guidance on career development. Students should be encouraged to reflect on their relationships with their parents and to cultivate a constructive, reciprocal form of filial piety. Encouraging blind obedience to parental expectations may undermine children’s career decision-making self-efficacy and autonomy, potentially resulting in regret if career choices are made without genuine personal reflection. For children, it is fundamental to enhance their self-efficacy in career decision-making and to seek parental support through the development of a constructive parent–child relationship grounded in RFP. When operating under conditions of AFP, children are encouraged to engage in reasonable negotiation with their parents, emphasizing the negative impact of AFP and advocating for a shift toward RFP. Such a transition may ultimately lead to a reduction in DBIPCG and promote healthier, more autonomous career decision-making.

Ultimately, our findings invite scholars and practitioners to reconceptualize filial piety as a dynamic construct that interacts with structural forces such as labor policies, social mobility pressures, and youth global outlooks. Career counselors and educators in collectivist societies should acknowledge the nuanced influence of AFP and RFP in shaping student career goals, while fostering supportive environments that encourage dialogue, emotional closeness, and shared decision-making. This shift may help mitigate the risks of intergenerational conflict and enable youth to pursue personally meaningful and culturally sensitive career paths.

### 6.3. Limitations and Future Directions

This study acknowledges multiple limitations. First, the data were attained through a self-report questionnaire, which could potentially introduce response biases. Second, caution is necessary when applying the findings to a broader population since the sample was confined to university/college students based in China. To enhance the extent to which future studies are conducted, it is recommended that upcoming research include individuals from diverse educational institutions, varying levels of education, and different academic fields. Third, the study did not explore the differences among students of different majors, nor many other individual factors. Specifically, this study did not examine the influence of demographic elements, including geographic location, family size, and participants’ rank among their siblings. For example, although the parental influence on career decision is vital, with globalization, Chinese students have increased autonomy and negotiation capabilities, which were not been examined in the present study. To address this limitation, it would be valuable to consider the impact of broader educational and demographic factors. Additionally, employing a mix of qualitative and quantitative methods for a more comprehensive analysis would be beneficial. Furthermore, the results were drawn from a group of students still enrolled in university or college with no job-search experience, and these factors shaped their life circumstances and career planning. Our understanding is incomplete without considering students with job-search or work experience. To overcome this, future research will include job-search or work experience participants, creating a control group for comparative analysis.

## Figures and Tables

**Table 1:** Descriptive statistics on all variables.

	Mean	SD	Skewness	Kurtosis	α
DBIPCG: ability	3.39	1.33	0.04	−0.48	0.91
DBIPCG: choice	3.11	1.44	0.19	−0.83	0.96
DBIPCG: enthusiasm	3.43	1.39	−0.01	−0.58	0.96
Career decision self-efficacy	6.27	2.00	0.16	−0.32	0.93
Parental support	3.56	0.90	−0.16	−0.23	0.97
Reciprocal filial piety	4.93	1.04	−1.37	2.63	0.96
Authoritative filial piety	3.13	1.33	0.53	0.40	0.94

*Note.* DBIPCG: discrepancies between individual-set and parent-set career goals.

**Table 2:** Confirmatory factor analysis on scales.

	χ^2^	df	χ^2^/df	CFI	TLI	SRMR
DBIPCG	413.81	86	4.8	0.93	0.91	0.05
Career decision self-efficacy	36.23	9	4.02	0.98	0.96	0.02
Parental support	182.89	52	3.52	0.96	0.95	0.03
Filial piety	371.53	101	3.68	0.94	0.92	0.09

*Note*. DBIPCG: discrepancies between individual-set and parent-set career goals.

**Table 3:** Correlation matrix on all variables.

Variable	1	2	3	4	5	6	7
1 DBIPCG: ability	——						
2 DBIPCG: choice	0.70 ***	——					
3 DBIPCG: enthusiasm	0.67 ***	0.79 ***	——				
4 Career decision self-efficacy	−0.02	0.07	0.02	——			
5 Parental support	−0.14 *	−0.26 ***	−0.14 *	0.31 ***	——		
6 Reciprocal filial piety	−0.06	−0.10	−0.04	0.17 **	0.68 ***	——	
7 Authoritative filial piety	0.40 ***	0.40 ***	0.23 ***	0.28 ***	0.25 ***	0.14 *	——

*Note:* DBIPCG: discrepancies between individual-set and parent-set career goals; * *p* < 0.05; ** *p* < 0.01; *** *p* < 0.001.

**Table 4:** Mediating effect testing of filial piety for the relationships between the career decision self-efficacy, parental support, and dimensions in discrepancies between individual-set and parent-set career goals (DBIPCG).

	Total Effects	Direct Effects	Indirect Effects via RFP	Indirect Effects via AFP
	[95%CI]	[95%CI]	[95%CI]	[95%CI]
Self-efficacy→ability	0.01 [−0.05, 0.08]	−0.04 [−0.10, 0.02]	−0.00 [−0.01, 0.00]	0.06 *** [0.02, 0.09]
Parental support→ability	−0.17 * [−0.31, −0.02]	−0.33 *** [−0.51, −0.16]	0.07 [−0.04, 0.19]	0.10 ** [0.03, 0.16]
Self-efficacy→choice	0.08 * [0.02, 0.14]	0.03 [−0.03, 0.08]	−0.00 [−0.01, 0.00]	0.05 *** [0.02, 0.09]
Parental support→choice	−0.30 *** [−0.44, −0.17]	−0.50 *** [−0.67, −0.34]	0.10 [−0.01, 0.22]	0.09 ** [0.03, 0.16]
Self-efficacy→enthusiasm	0.04 [−0.03, 0.10]	0.01 [−0.06, 0.07]	−0.00 [−0.01, 0.00]	0.03 ** [0.01, 0.06]
Parental support→enthusiasm	−0.18 * [−0.32, −0.03]	−0.31 *** [−0.50, −0.13]	0.08 [−0.04, 0.20]	0.06 * [0.01, 0.10]

*Note*: * *p* < 0.05; ** *p* < 0.01; *** *p* < 0.001.

## Data Availability

Data are available upon request.

## References

[B1-behavsci-15-01135] Akosah-Twumasi P., Emeto T. I., Lindsay D., Tsey K., Malau-Aduli B. S. (2018). A systematic review of factors that influenceyouths career choices—The role of culture. Frontiers in education.

[B2-behavsci-15-01135] Alliman-Brissett A. E., Turner S. L., Skovholt T. M. (2004). Parent support and African American adolescents’ career self-efficacy. Professional School Counseling.

[B3-behavsci-15-01135] Anderson K. L., Mounts N. S. (2012). Searching for the self: An identity control theory approach to triggers of occupational exploration. The Journal of Genetic Psychology.

[B4-behavsci-15-01135] Bandura A. (1977). Self-efficacy: Toward a unifying theory of behavioral change. Psychological Review.

[B5-behavsci-15-01135] Bandura A. (1986). Social foundations of thought and action: A social cognitive theory *(pp. xiii, 617)*.

[B6-behavsci-15-01135] Bandura A. (2002). Social cognitive theory in cultural context. Applied Psychology.

[B7-behavsci-15-01135] Bandura A., Locke E. A. (2003). Negative self-efficacy and goal effects revisited. The Journal of Applied Psychology.

[B8-behavsci-15-01135] Bedford O., Yeh K.-H. (2019). The history and the future of the psychology of filial piety: Chinese norms to contextualized personality construct. Frontiers in Psychology.

[B9-behavsci-15-01135] Bedford O., Yeh K.-H. (2021). Evolution of the conceptualization of filial piety in the global context: From skin to skeleton. Frontiers in Psychology.

[B10-behavsci-15-01135] Biesanz J. C., Falk C. F., Savalei V. (2010). Assessing mediational models: Testing and interval estimation for indirect effects. Multivariate Behavioral Research.

[B11-behavsci-15-01135] Chen W.-W. (2014). The relationship between perceived parenting style, filial piety, and life satisfaction in Hong Kong. Journalof Family Psychology.

[B12-behavsci-15-01135] Clements A. J., Kinman G., Leggetter S., Teoh K., Guppy A. (2016). Exploring commitment, professional identity, andsupport for student nurses. Nurse Education in Practice.

[B13-behavsci-15-01135] Constantine M. G., Wallace B. C., Kindaichi M. M. (2005). Examining contextual factors in the career decision status of African American adolescents. Journal of Career Assessment.

[B14-behavsci-15-01135] Cross S. E., Bacon P. L., Morris M. L. (2000). The relational-interdependent self-construal and relationships. Journal of Personality and Social Psychology.

[B15-behavsci-15-01135] Donovan J. J. (2009). Antecedents of discrepancy production in an achievement setting. Journal of Managerial Issues.

[B16-behavsci-15-01135] Edwards K., Quinter M. (2011). Factors influencing students career choices among secondary school students in Kisumu municipality, Kenya. Journal of Emerging Trends in Educational Research and Policy Studies (JETERAPS).

[B17-behavsci-15-01135] Feldman R. (2007). Parent–infant synchrony and the construction of shared timing; physiological precursors, developmental outcomes, and risk conditions. Journal of Child Psychology and Psychiatry.

[B18-behavsci-15-01135] Gagnon É., Ratelle C. F., Guay F., Duchesne S. (2019). Developmental trajectories of vocational exploration from adolescence to early adulthood: The role of parental need supporting behaviors. Journal of Vocational Behavior.

[B19-behavsci-15-01135] Gibbons M. M., Borders L. D. (2010). Prospective first-generation college students: A social-cognitive perspective. The Career Development Quarterly.

[B20-behavsci-15-01135] Guo X., Li J., Niu Y., Luo L. (2022). The relationship between filial piety and the academic achievement and subjective wellbeing of Chinese early adolescents: The moderated mediation effect of educational expectations. Frontiers in Psychology.

[B21-behavsci-15-01135] Hackett G., Betz N. E. (1981). A self-efficacy approach to the career development of women. Journal of Vocational Behavior.

[B22-behavsci-15-01135] Hampton N. Z. (2005). Testing for the structure of the career decision self-efficacy scale-short form among Chinese college students. Journal of Career Assessment.

[B23-behavsci-15-01135] He A., Yao Y. (2018). The relationship between social support, psychological capital and employability of college. Journal of Xinyang Normal University (Philosophy and Social Sciences Edition).

[B24-behavsci-15-01135] Hollenbeck J. R., Williams C. R., Klein H. J. (1989). An empirical examination of the antecedents of commitment to difficult goals. Journal of Applied Psychology.

[B25-behavsci-15-01135] Hou Z.-J., Leung S. A. (2011). Vocational aspirations of Chinese high school students and their parents’ expectations. Journal of Vocational Behavior.

[B26-behavsci-15-01135] Kantamneni N., Dharmalingam K., Orley G., Kanagasingam S. K. (2018). Cultural factors, perceived barriers, and Asian American career development: An application of social cognitive career theory. Journal of Career Assessment.

[B27-behavsci-15-01135] Kemph J. P. (1969). Erik H. Erikson. Identity, youth and crisis. New York: W. W. Norton Company, 1968. Behavioral Science.

[B28-behavsci-15-01135] Klein H. J., Molloy J. C., Brinsfield C. T. (2012). Reconceptualizing workplace commitment to redress a stretched construct: Revisiting assumptions and removing confounds. The Academy of Management Review.

[B29-behavsci-15-01135] Kush K., Cochran L. (1993). Enhancing a sense of agency through career planning. Journal of Counseling Psychology.

[B30-behavsci-15-01135] Lapan R. T., Hinkelman J. M., Adams A., Turner S. (1999). Understanding rural adolescents’ interests, values, and efficacy Expectations. Journal of Career Development.

[B31-behavsci-15-01135] Latham G. (2012). Work motivation: History, theory, research, and practice.

[B32-behavsci-15-01135] Latham G. P., Locke E. A. (1979). Goal setting—A motivational technique that works. Organizational Dynamics.

[B33-behavsci-15-01135] Latham G. P., Yukl G. A. (1976). Effects of assigned and participative goal setting on performance and job satisfaction. Journal of Applied Psychology.

[B34-behavsci-15-01135] Lent R. W., Brown S. D. (2006). On conceptualizing and assessing social cognitive constructs in career research: A measurement guide. Journal of Career Assessment.

[B35-behavsci-15-01135] Lent R. W., Brown S. D., Hackett G. (1994). Toward a Unifying social cognitive theory of career and academic interest, choice, and performance. Journal of Vocational Behavior.

[B36-behavsci-15-01135] Lent R. W., Brown S. D., Hackett G. (2000). Contextual supports and barriers to career choice: A social cognitive analysis. Journal of Counseling Psychology.

[B37-behavsci-15-01135] Lent R. W., Sheu H.-B., Brown S. D. (2010). The self-efficacy—Interest relationship and RIASEC type: Which is figure and which is ground? Comment on Armstrong and Vogel (2009). Journal of Counseling Psychology.

[B38-behavsci-15-01135] Leong F. T. L., Hardin E. E., Gupta A. (2011). Self in vocational psychology: A cultural formulation approach. Developing self in work and career: Concepts, cases, and contexts.

[B39-behavsci-15-01135] Leung S. A., Hou Z.-J., Gati I., Li X. (2011). Effects of parental expectations and cultural-values orientation on career decision-making difficulties of Chinese University students. Journal of Vocational Behavior.

[B40-behavsci-15-01135] Li X., Zou H., Liu Y., Zhou Q. (2014). The relationships of family socioeconomic status, parent–adolescent conflict, and filial piety to adolescents’ family functioning in mainland China. Journal of Child and Family Studies.

[B41-behavsci-15-01135] Lian Z., Feng N. (2021). Job value and organizational socialization of the youth of Guangdong-Hong Kong-Macao Greater Bay Area: The mediation of career exploration?. Scientific Programming.

[B42-behavsci-15-01135] Liu J., McMahon M., Watson M. (2015). Parental influence on mainland Chinese children’s career aspirations: Child and parental perspectives. International Journal for Educational and Vocational Guidance.

[B43-behavsci-15-01135] Locke E. A., Latham G. P. (1990). Work motivation and satisfaction: Light at the end of the tunnel. Psychological Science.

[B44-behavsci-15-01135] Locke E. A., Latham G. P. (2002). Building a practically useful theory of goal setting and task motivation: A 35-year odyssey. American Psychologist.

[B45-behavsci-15-01135] Locke E. A., Latham G. P. (2006). New directions in goal-setting theory. Current Directions in Psychological Science.

[B46-behavsci-15-01135] Love J., Selker R., Marsman M., Jamil T., Dropmann D., Verhagen J., Ly A., Gronau Q. F., Šmíra M., Epskamp S., Matzke D., Wild A., Knight P., Rouder J. N., Morey R. D., Wagenmakers E.-J. (2019). JASP: Graphical statistical software for common statistical designs. Journal of Statistical Software.

[B47-behavsci-15-01135] Ma P.-W. W., Yeh C. J. (2005). Factors influencing the career decision status of Chinese American youths. The Career Development Quarterly.

[B48-behavsci-15-01135] Ma Y., Siu A., Tse W. S. (2018). The role of high parental expectations in adolescents’ academic performance and depression in Hong Kong. Journal of Family Issues.

[B49-behavsci-15-01135] Onifade E., Lee J., Mennicke A., Holmes J. L., Harris R. (2016). Predicting delinquent behaviors for Korean youth using the parent–child relationship and career goal tension. Journal of Ethnicity in Criminal Justice.

[B50-behavsci-15-01135] Pinquart M., Ebeling M. (2020). Parental educational expectations and academic achievement in children and adolescents—A meta-analysis. Educational Psychology Review.

[B51-behavsci-15-01135] Rosseel Y. (2012). lavaan: An R package for structural equation modeling. Journal of Statistical Software.

[B52-behavsci-15-01135] Rutherford T. (2015). Emotional well-being and discrepancies between child and parent educational expectations and aspirations in middle and high school. International Journal of Adolescence and Youth.

[B53-behavsci-15-01135] Ryan R. M., Deci E. L. (2018). Self-determination theory: Basic psychological needs in motivation, development, and wellness.

[B54-behavsci-15-01135] Salim R. M. A., Samosir E., Rumalutur N. A., Situmorang D. D. B. (2024). The role of core self-evaluation as a moderator in the relationship between perceived parental support and career adaptability among university students from papua region. International Journal for the Advancement of Counselling.

[B55-behavsci-15-01135] Samsuri A. S. B., Arifin T. R. B. T., Hussin S. B. (2016). Perception of undergraduate accounting students towards professional accounting career. International Journal of Academic Research in Accounting, Finance and Management Sciences.

[B56-behavsci-15-01135] Sawitri D. R., Creed P. A. (2017). Collectivism and perceived congruence with parents as antecedents to career aspirations: A social cognitive perspective. Journal of Career Development.

[B57-behavsci-15-01135] Sawitri D. R., Creed P. A., Perdhana M. S. (2021). The discrepancies between individual-set and parent-set career goals scale: Development and initial validation. Journal of Career Development.

[B58-behavsci-15-01135] Schoon I., Polek E. (2011). Teenage career aspirations and adult career attainment: The role of gender, social background and general cognitive ability. International Journal of Behavioral Development.

[B59-behavsci-15-01135] Song H. (2021). Raising cosmopolitan children: Chinese middle-class parents’ educational strategies. Comparative Education.

[B60-behavsci-15-01135] Song Y., Mu F., Zhang J., Fu M. (2022). The Relationships between career-related emotional support from parents and teachers and career adaptability. Frontiers in Psychology.

[B61-behavsci-15-01135] Steers R. M., Porter L. W. (1974). The role of task-goal attributes in employee performance. Psychological Bulletin.

[B62-behavsci-15-01135] Sun P., Yang Z., Jiang H., Chen W., Xu M. (2023). Filial piety and meaning in life among late adolescents: A moderated mediation model. Children and Youth Services Review.

[B63-behavsci-15-01135] Tang M. (2002). A comparison of Asian American, Caucasian American, and Chinese college students: An initial report. Journal of Multicultural Counseling and Development.

[B64-behavsci-15-01135] Tynkkynen L., Nurmi J.-E., Salmela-Aro K. (2010). Career goal-related social ties during two educational transitions: Antecedents and consequences. Journal of Vocational Behavior.

[B65-behavsci-15-01135] Van Wyk E. (2011). A note: The SAICA part I qualifying examinations: Factors that may influence candidates’ success. South African Journal of Accounting Research.

[B66-behavsci-15-01135] Wan J., Zhang Q., Mao Y. (2024). How filial piety affects Chinese college students’ social networking addiction—A chain-mediated effect analysis. Computers in Human Behavior Reports.

[B67-behavsci-15-01135] Wang Q., Pomerantz E. M., Chen H. (2007). The role of parents’ control in early adolescents’ psychological functioning: A longitudinal investigation in the United States and China. Child Development.

[B68-behavsci-15-01135] Wei H., Wei Y., He A., Duan H., Liu Y. (2019). Effect of just world belief on internet addiction: A moderated mediation model. Chinese Journal of Clinical Psychology.

[B69-behavsci-15-01135] Wells P. K. (2013). Mirror, mirror on the wall.

[B70-behavsci-15-01135] Wilk P., Clark A. F., Maltby A., Tucker P., Gilliland J. A. (2018). Exploring the effect of parental influence on children’s physical activity: The mediating role of children’s perceptions of parental support. Preventive Medicine.

[B71-behavsci-15-01135] Xiao Q., Liang X., Liu L., Klarin A., Zhang C. (2023). How do work–Life balance programmes influence nurses’ psychological well-being? The role of servant leadership and learning goal orientation. Journal of Advanced Nursing.

[B72-behavsci-15-01135] Xu C. (2012). Study on the relationship of social support, psychological capital and career decision-making difficulty.

[B73-behavsci-15-01135] Yao J., Wei X., Geng Q., Sun X., Xu H., Qi Y. (2015). Mediating effect of self-perceived social competence and self-esteem between dual filial piety and life satisfaction in high school students. Chinese Mental Health Journal.

[B74-behavsci-15-01135] Yeh K.-H. (2004). Filial piety and autonomous development of Chinese adolescents. The family and social change in Chinese societies.

[B75-behavsci-15-01135] Yeh K.-H., Bedford O. (2003). A test of the Dual Filial Piety model. Asian Journal of Social Psychology.

[B76-behavsci-15-01135] Zhang Y., Guan C., Jiang J., Zhu C., Hu X. (2023). Mediating effect of resilience on the relationship between perceived social support and burnout among Chinese palliative nurses. Journal of Clinical Nursing.

[B77-behavsci-15-01135] Zhao M., Tong Y. (2025). Beyond the paycheck: Family systems, adult children’s job sector, and parental subjective well-being in China. Journal of Marriage and Family.

